# Supplementary material for: Controlling piezoresistance in single molecules through the isomerisation of bullvalenes
Source: Nat Commun. 2023 Oct 3;14:6089. doi: 10.1038/s41467-023-41674-z (PMC10547723; doi:10.1038/s41467-023-41674-z)
Supplement: Supplementary file 1 — Supplementary Information [file 41467_2023_41674_MOESM1_ESM.pdf]

Supplementary Information

Controlling Piezoresistance in Single Molecules through the Isomerisation of Bullvalenes

Jeffrey R. Reimers[1,2*], *Tiexin Li*[3], *André P. Birvé*[4], *Likun Yang*[1], *Albert C. Aragonès*[5,6],

Thomas Fallon[4*], *Daniel S. Kosov*[7*], *and Nadim Darwish*[3*]

1. International Centre for Quantum and Molecular Structures and the Department of Physics, Shanghai University, Shanghai 200444, China.
2. School of Mathematical and Physical Sciences, University of Technology Sydney, Sydney, NSW 2007, Australia
3. School of Molecular and Life Sciences, Curtin University, Bentley, WA 6102, Australia.
4. School of Environmental and Life Sciences, University of Newcastle, Callaghan, NSW 2308, Australia
5. Department of Materials Science and Physical Chemistry, University of Barcelona, Marti i Franquès 1, 08028 Barcelona, Catalonia (Spain).
6. Institute of Theoretical and Computational Chemistry, University of Barcelona, Diagonal 645, 08028 Barcelona, Catalonia (Spain).
7. College of Science and Engineering, James Cook University, Townsville, QLD 4811, Australia.

Contents

Supplementary Figures .... **Fehler! Textmarke nicht definiert.**

Supplementary Note 1. STMBJ Blinking approach experiments and correlation maps 5

Supplementary Note 2. Conductance trace simulations using the 4-atom tip model 6

Supplementary Figures

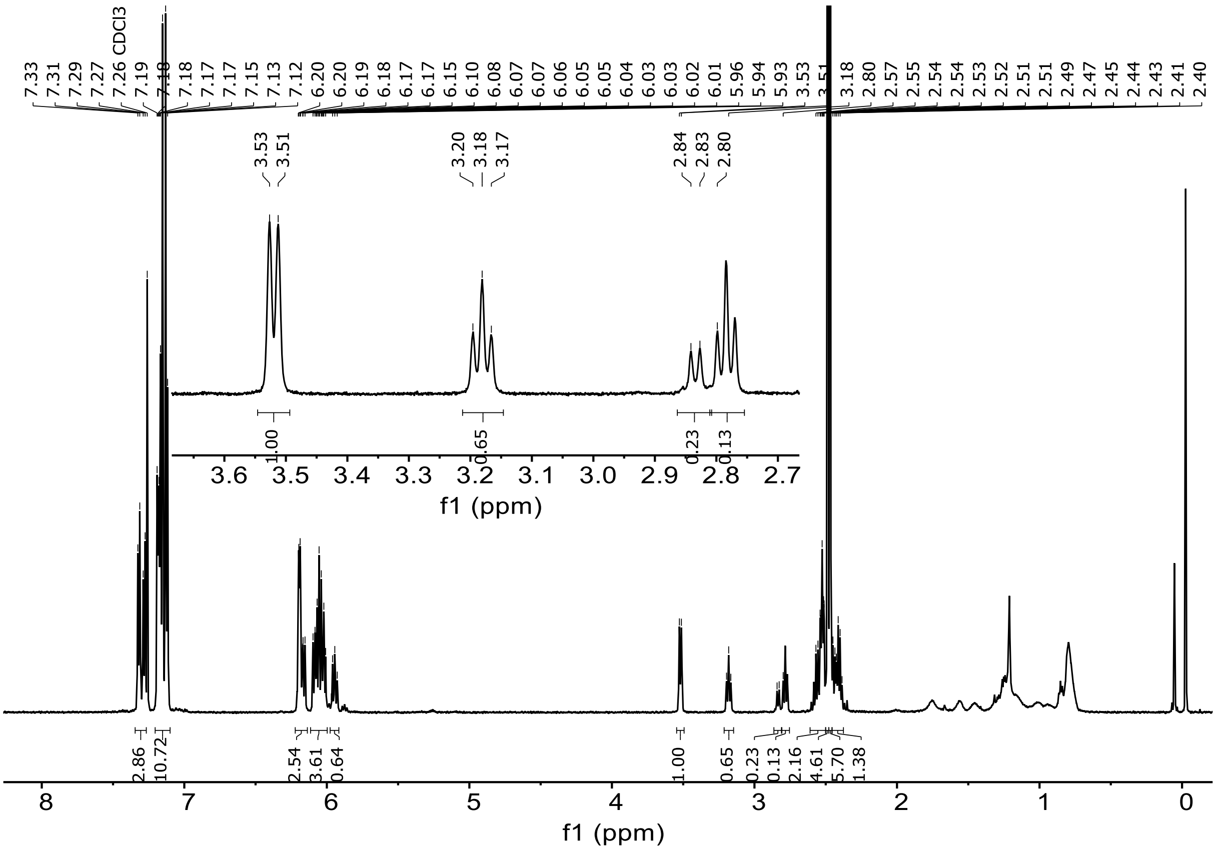

Supplementary Figure 1 | ^{1}H NMR spectrum of bis(4-thioanisole)bullvalene at -50 °C.

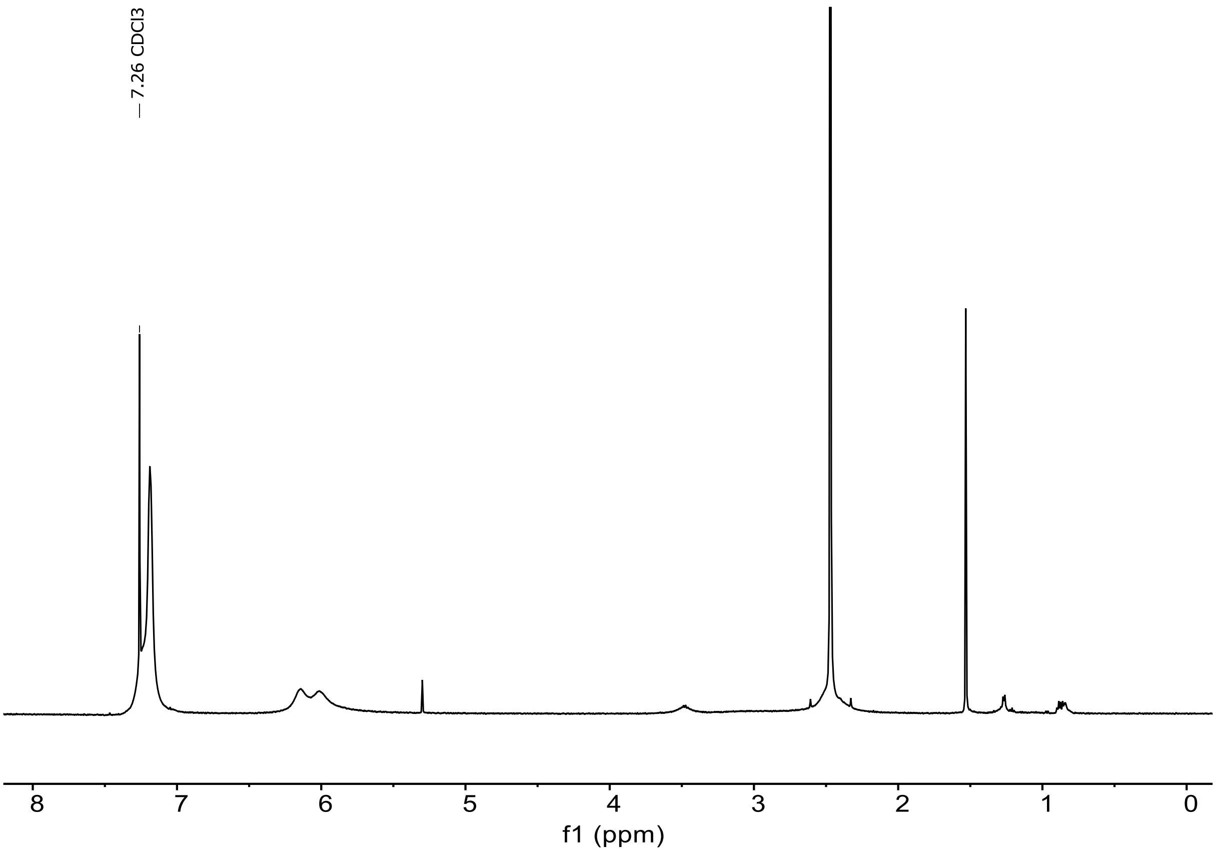

Supplementary Figure 2 | ^{1}H NMR spectrum of bis(4-thioanisole)bullvalene at 25 °C.

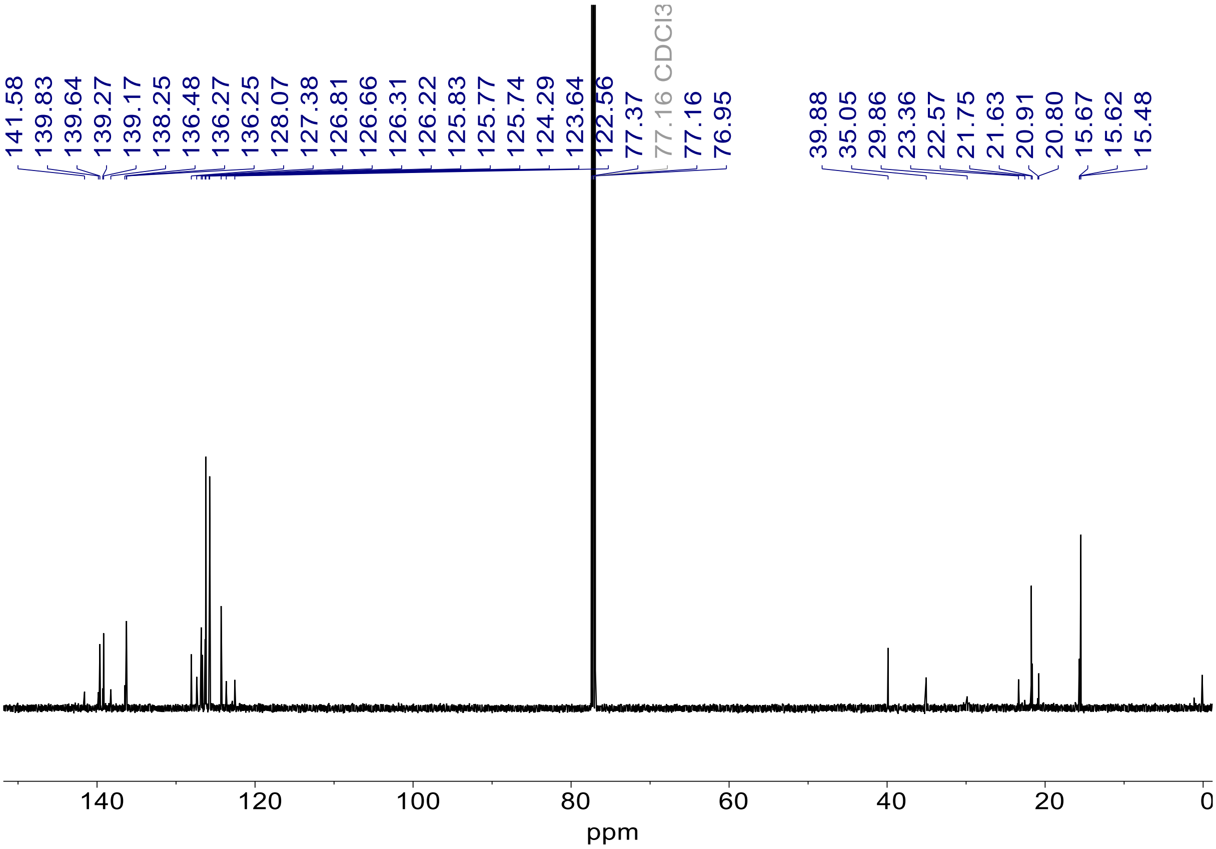

Supplementary Figure 3 | ^{13}C NMR spectrum of bis(4-thioanisole)bullvalene at –50 °C.

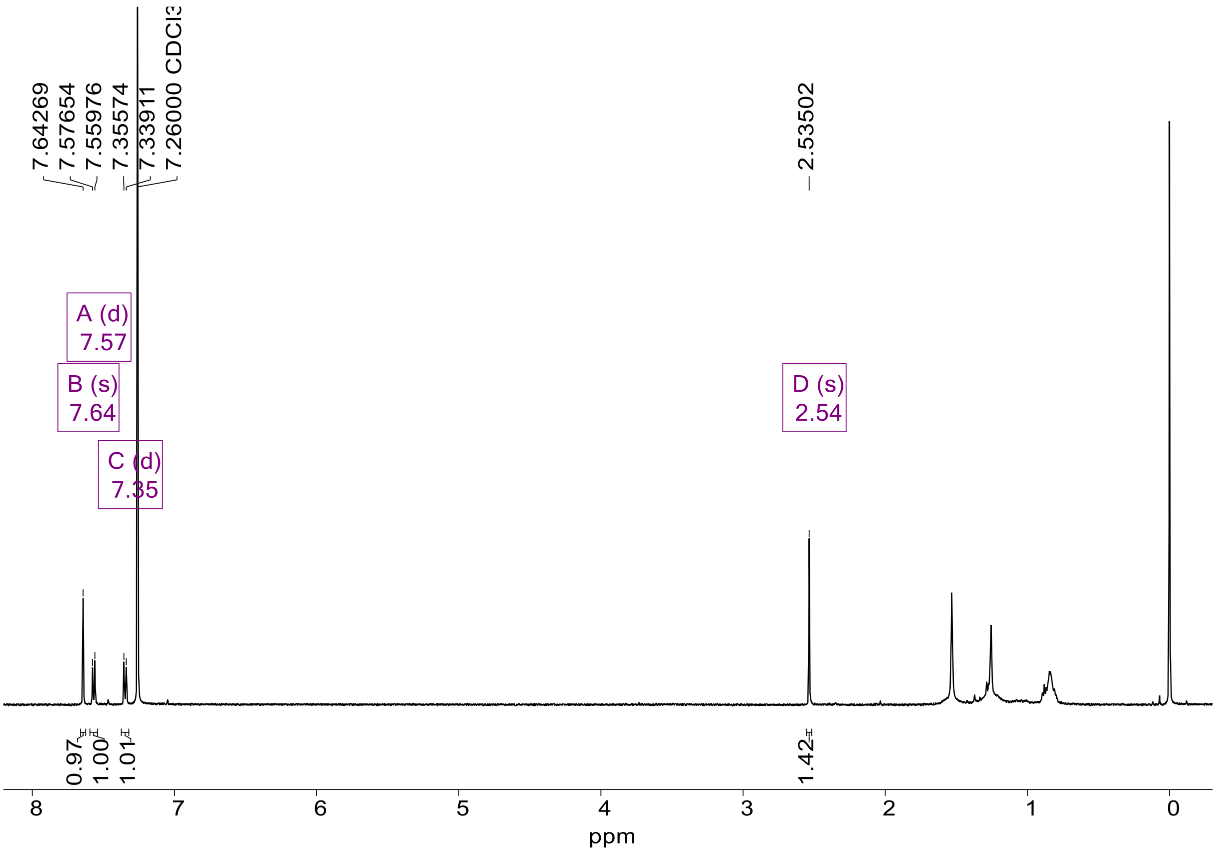

Supplementary Figure 4 | ^{1}H NMR spectrum of para-(4,4'-bi(methylthio)terphenyl.

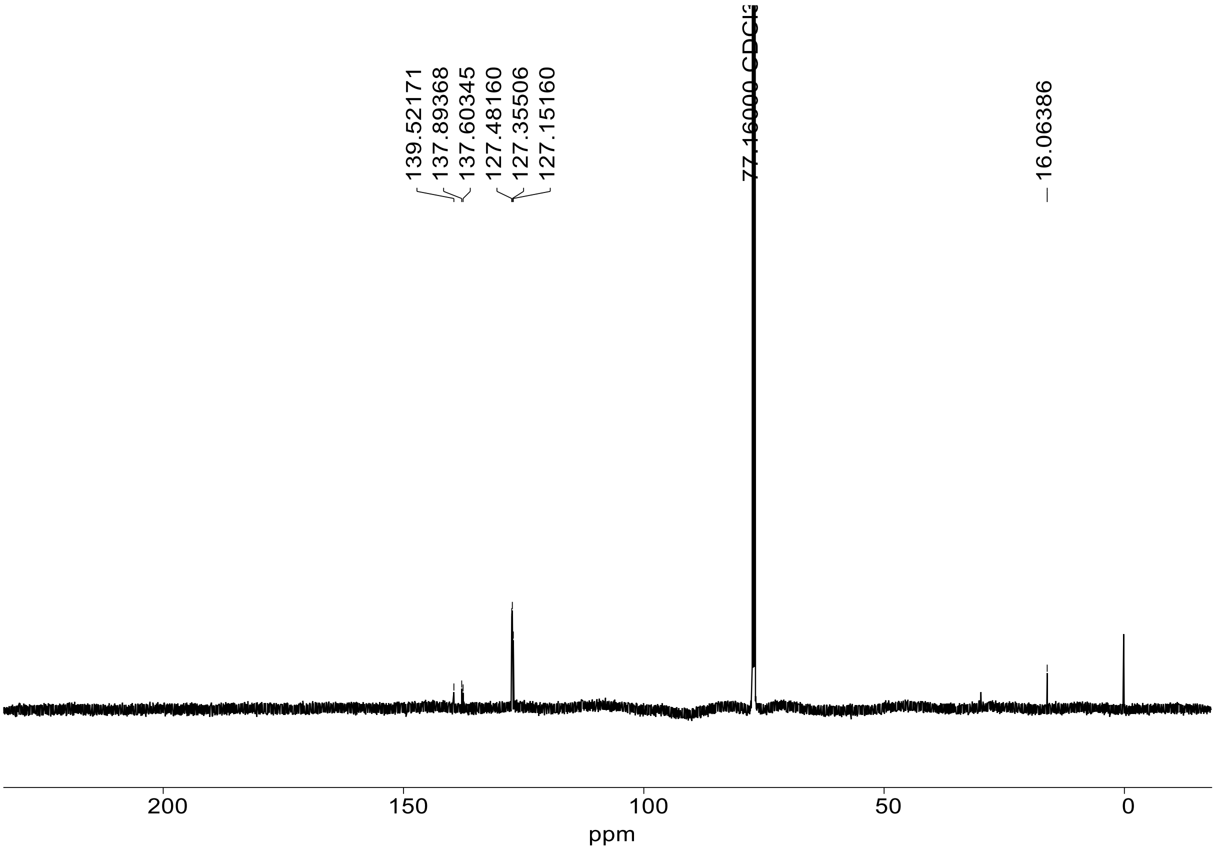

Supplementary Figure 5 | ^{13}C NMR spectrum of para-(4,4'-bi(methylthio)terphenyl.

Supplementary Note 1. STMBJ blinking approach experiments and correlation maps

In the blinking approach, the STM tip is fixed at a specific distance from the surface in the presence of a dilute concentration (5 μM) of the bullvalene molecules in 1,2,4–trichlorobenzene as the solvent. To determine the distance between the two Au electrodes, a specific set-point tunnelling current is first chosen through the STM feedback loop, and the tunnelling current decay β is determined by measuring the current decay for a defined distance. In the absence of molecules, the current decay values obtained were ≈ 7 nm^{-1}, and the electrode–electrode gap distance can be evaluated using the equation (1) $G = G_0 e^{-\beta d}$ where d is the distance separating the two electrodes and (2) $G_0 = 2e^2/h$ is the quantum of conductance. Given this, an estimate can be made for the junction separation from a measured value of the set-point current at a specific voltage bias.

Errors in the determination of electrode–electrode distance could arise if a high concentration of the target molecule is used, because the decay rate of the current with increasing distance can be reduced if a self-assembled monolayer forms inside the junction. To prevent such a monolayer forming, ensuring that at most one isolated molecule is in the junction region at a time, a low concentration of 5 μM of bullvalene molecules was used in the blinking experiments.

The blinking method differs significantly from the STMBJ experiment in that no physical contact occurs between the STM tip and the Au(111) surface, a fact that minimises possible tip-induced artefacts. In these experiments, the electrode–electrode gap d is set using the STM control electronics to bring the STM tip close enough to the surface to achieve the desired tunnelling current. Once this distance is established, the feedback system is disabled. Occasionally, a bullvalene molecule bridges between the tip and the surface, and this occurrence is accompanied by a sudden jump in current referred to as 'the blink".

We built the correlation (conductance) map following previous procedures.[1] An automated algorithm fragments the captured conductance range in different regions (*y*-axis bins). The algorithm analyses the captures inside the specific bin only and, therefore, only the counts of the current decays containing plateaus inside the region are accumulated and plotted in the *x*-axis. The key point of this methodology is that when a plateau is detected and the complete current trace contains more plateaus at conductance different to the one studied, they are accumulated in the 2D map, thus highlighting the interdependence between the occurrences of the different current plateaus.

Supplementary Note 2. Conductance trace simulations using the 4-atom tip model

The rate of isomerisaton from isomer X to Y is given by the Arrhenius equation:

$$\frac{d[X]}{dt} = A \exp\left(\frac{-\Delta E_{XY}^{\ddagger}}{\mathrm{k_B}T}\right)[X] \quad (3)$$

where $\Delta E_{XY}^{\ddagger}$ is the PBE-D3BJ calculated activation energy (difference in energies between those in Figure 5c and those in Figure 5b), T = 300 K is the temperature, and A is determined such that the analogously calculated rate for isomerisation of bullvalene in solution agrees with the observed value. Solution of the coupled equations for all 20 constitutional and conformational isomers considered yields the mole fractions shown in Figure 5f,g. These kinetics simulations were performed using a time stop of 11 ns, with faster reactions that this being taken to be in equilibrium. Also, the kinetics equations were solved applying a tip retraction rate of 0.5 Å ms^{-1} that corresponds to the value used in the experiments.

To simulate individual conductance traces, the mole fraction at any instant in time must be constrained to depict a single isomer, i.e., instantaneous mole fractions can be only 0 or 1. To achieve this, the probabilities of reaction at each instant in time are obtained from equations of the above form, and then a random number is chosen and used to determine the composition at the next time instant. In these simulations, each "instant in time" is taken to last for a timestep of 11 ns, and 3000 such time steps are then sampled and the current averaged. This average is therefore performed over the sampling time of 0.033 ms used in the experiments to make each individual current measurement. In this process, all isomerisation reactions that occur on a timescale faster than 0.033 ms are averaged over. In general, the observed conductance in each individual measurement is found to arise from averages over multiple isomers. Initially, the $\mathbf{A_{mm}}$ and its symmetrically equivalent $\mathbf{A_{pp}}$ conformer are predicted to dominate the conductance, but subsequently the small amount of $\mathbf{B_{pm}}$ found on average to be present in each 0.033 ms time interval is predicted to dominate (Figure 5f,h).

A large number of simulated conductance traces can then be averaged, resulting in average isomer mole fractions that converge towards the directly simulated results shown in Figure 5f,g. This is demonstrated in Supplementary Figure 6 in which the results from averaging over 3000 traces (Supplementary Figure 6b) are shown and compared to those from Figure 5 that are reproduced in Supplementary Figure 6a. In contrast, averaging the conductance using the easily determined average mole fractions from Figure 5f and 5g, shown in Supplementary Figure 7a, does not lead to the same conductance histogram as obtained from averaging the traces shown in Figure 5h (and reproduced in Supplementary Figure 7b). This is because utilisation of the average mole fraction in this way does not take into account the fact that the current is averaged only over 0.033 ms intervals during each measurement. Using the average mole fractions assumes that each individual trace represents complete chemical equilibrium, whereas many reactions are too slow for this to occur (see Figure 5d).

Results from conductance-trace simulations are also shown in Supplementary Figures 6-8 obtained by varying the calculated isomer energy differences and transition-state energies in simple ways. One set of results involves adding 0.1 eV to all transition-state energies. The second involves stabilizing all **A** conformers by 0.1 eV, with all related transition states stabilised by half this amount, as well as analogously destabilizing all **C** isomers and transition states by the same amounts. The third variation involves destabilizing all **C** and **D** conformers by 0.1 eV, with again the associated transition states destabilised by 0.05 eV. Supplementary Figure 8 shows that all three of these variations significantly inhibits reverse reactions, predicting conductance traces in much better agreement with the observed traces. Also, both

the original simulated histogram and those arising from the three variations display two sharp peaks in the low-conductance region (Supplementary Figure 7), in good agreement with experiment (Figure 3c). In each case, the two peaks are attributed to variations in the small amount (Supplementary Figure 6) of the highly conductive (Figure 5e) isomer $\mathbf{B_{pm}}$ found in the dynamic equilibria established on the 0.033 ms experimental timescale.

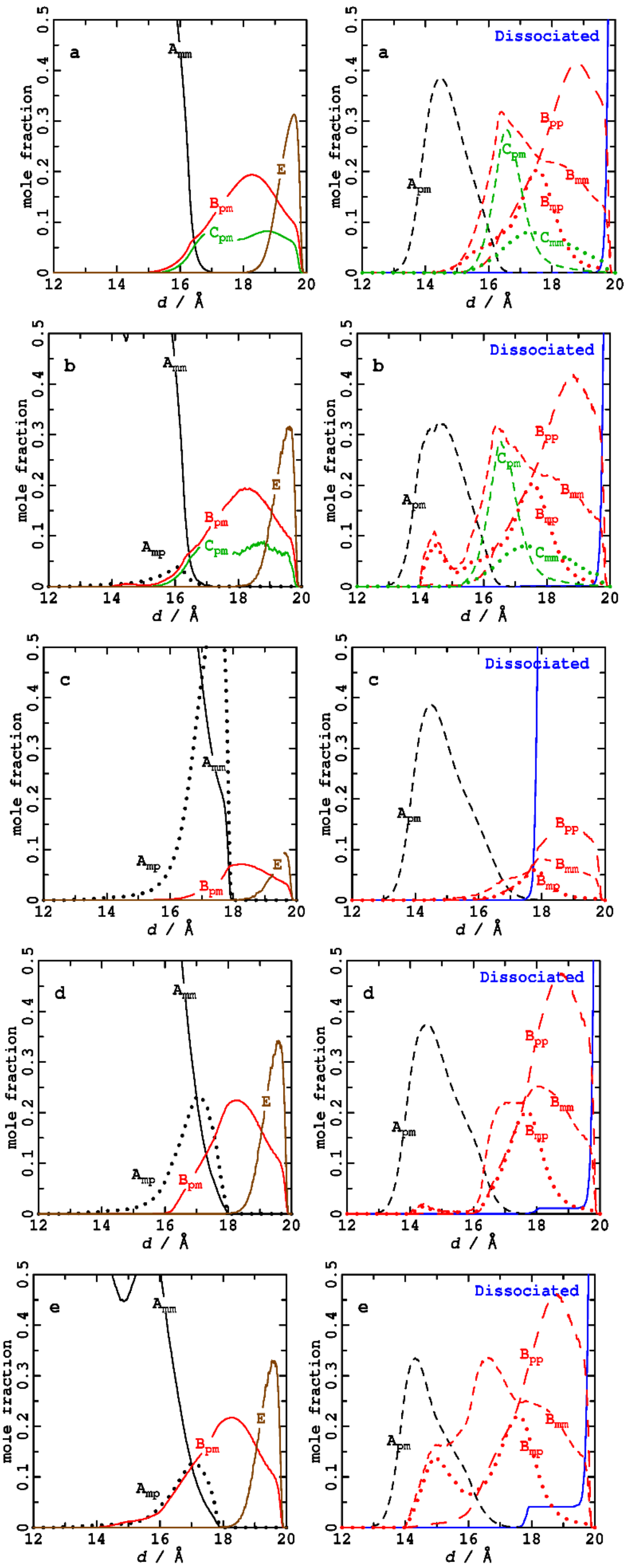

Supplementary Figure 6 | Mole fractions simulated directly and from trace averaging. a. reproduces the results shown in Figure 5f,g, obtained by direct solution of the kinetics equations. **b**. numerical simulation of the results in **a** obtained by averaging 3000 simulated conductance traces (see Supplementary Figure 8), demonstrating reasonable convergence. **c**. From 3000 traces, increasing all activation energies by 0.10 eV. **d**. From 3000 traces, stabilising all **A** conformers by 0.1 eV and destabilizing all **C** conformers by 0.1 eV, and associated activation energies by half of these changes. **e**. From 3000 traces, destabilising all **C** and **D** conformers by 0.10 eV and associated activation energies by 0.05 eV.

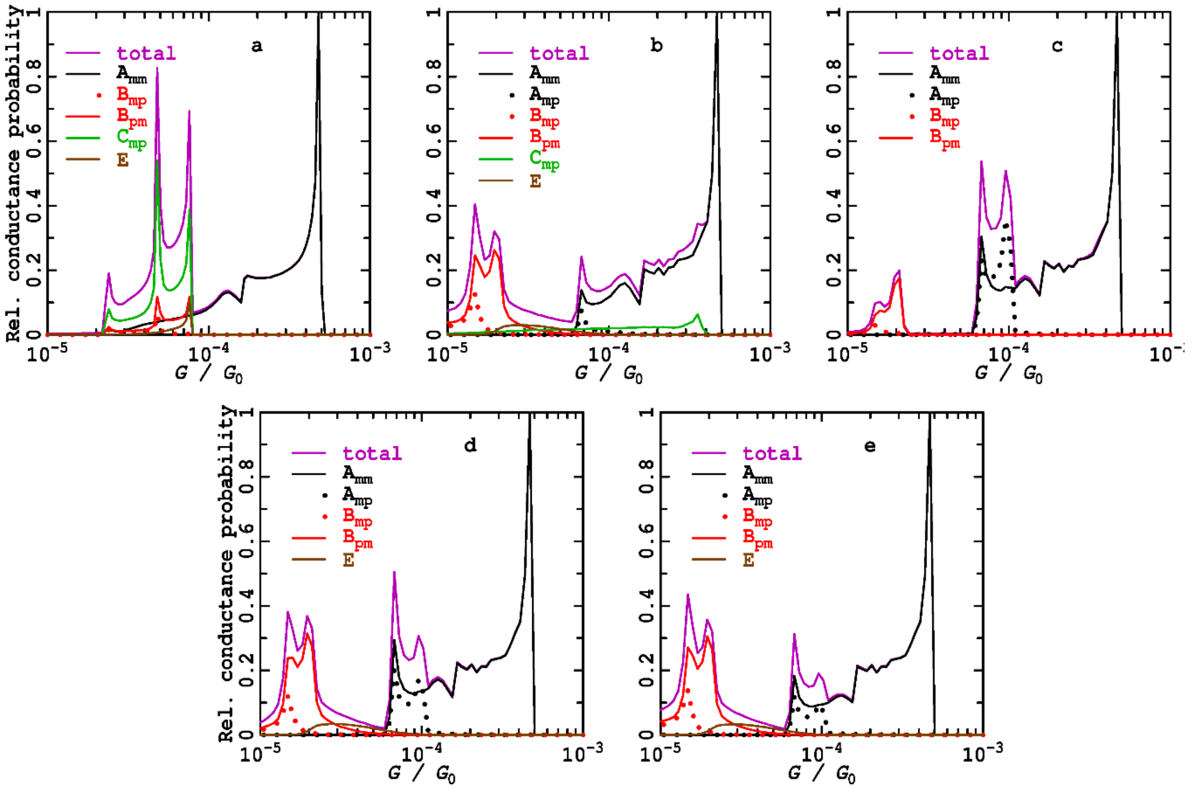

Supplementary Figure 7 | Conductance histograms evaluated by different methods. a. obtained by direct solution of the kinetics equations, hence making the assumption of extensive time averaging during the sampling time. **b**. obtained by averaging 3000 simulated conductance traces (see Supplementary Figure 8) with 0.033 ms sampling time, reproducing results from Figure 5h. **c**. From 3000 traces, increasing all activation energies by 0.1 eV. **d**. From 3000 traces, stabilising all **A** conformers by 0.1 eV and destabilizing all **C** conformers by 0.1 eV, and associated activation energies by half of these changes. **e**. From 3000 traces, destabilising all **C** and **D** conformers by 0.10 eV, and associated activation energies by 0.05 eV. Source data are provided as a Source Data file.

The simulations performed by increasing all transition-state energies by 0.1 eV differ from the others in that 68% of the traces show **A** conformers dissociating from the gold contacts before isomerisation to **B** occurs. This is in qualitative agreement with experiment, in which over 90% of the traces did not depict isomerisation to low-conductance forms; quantitative agreement with this experimental feature can be obtained by enhancing the transition-state energy increase to 0.13 eV. In these simulations, the **A** to **D** transition-state energy is increased such that isomerisation becomes slow on the ≈3 ms time scale need to go from separations at which the reaction can occur to separations at which dissociation can occur.

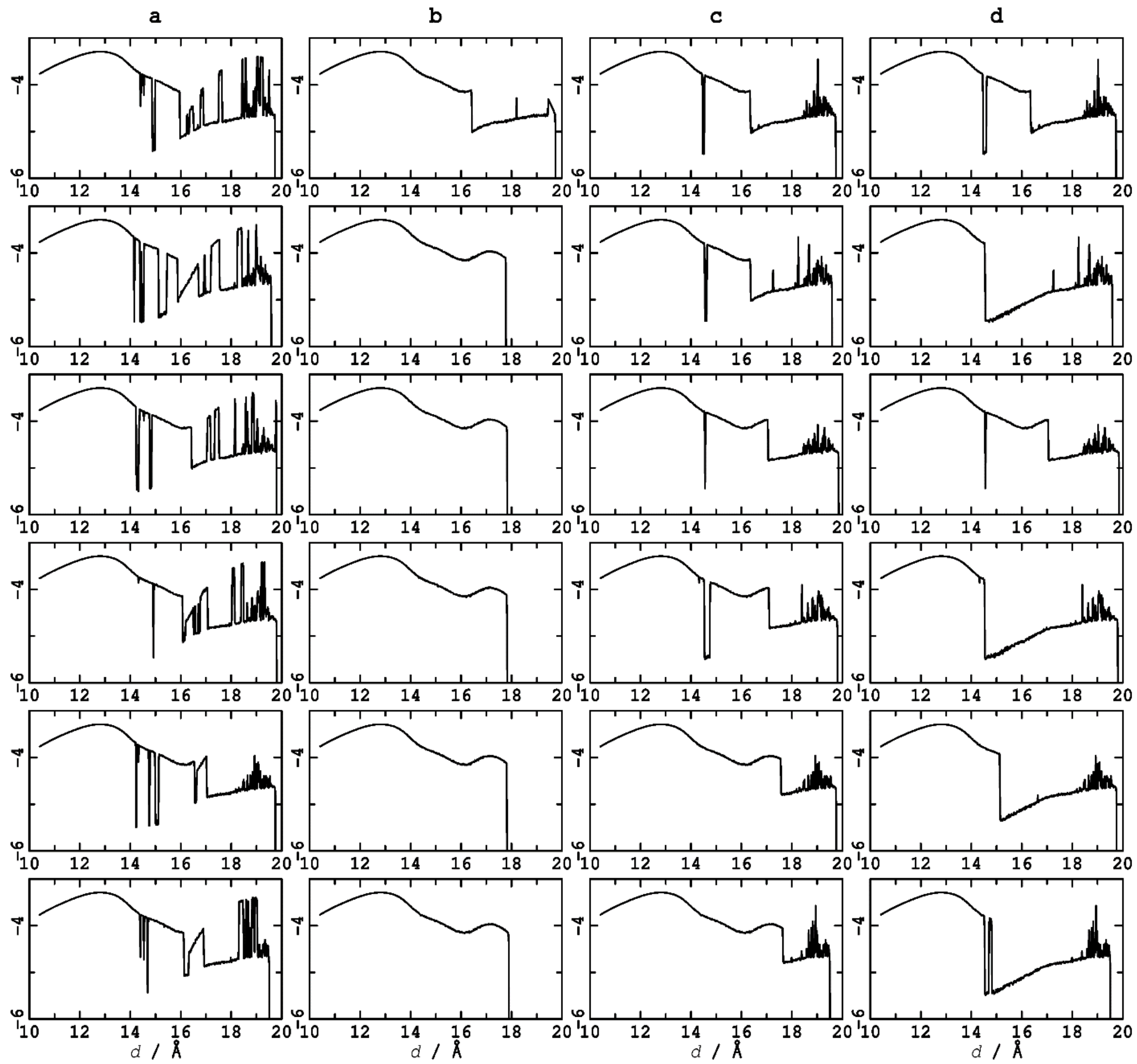

Supplementary Figure 8 | Six example simulated conductance traces. a. using the orignal DFT-calculated reaction energies and activation energies. **b**. increasing all activation energies by 0.1 eV. **c**. stabilising all **A** conformers by 0.1 eV and destabilizing all **C** conformers by 0.1 eV, and associated activation energies by half of these changes. **d**. destabilising all **C** and **D** conformers by 0.1 eV, and associated activation energies by 0.05 eV.

The likelihood that the calculations underestimate the energy of the **A** to **D** transition state by 0.13 eV can be gauged by examining the reactant, transition-state and product structures presented in Supplementary Figure 9 for d = 16.4 Å. In this figure, atoms involved in Cope rearrangements are highlighted in green. At the structures of interest, the junction is placed under tension by the tip retraction. The transition state is early, corresponding closely to the structure of the reactant $\mathbf{A}_{\mathbf{mm}}$. This processes lengthens the bond in the forming 3-membered ring that lies in the direction of the tensile force, and allows a net 0.06 Å extension in the distance between the two bridgehead aryl carbon atoms that bind to the bullvalene. Hence the applied tension acts to stabilise the transition state, as reflected in a net change of the activation energy of the reaction by –0.03 eV. This stabilisation leads to the four-fold increase in reaction rate shown for this tip extension in Figure 5d. Subsequent reaction from the transition state to yield the product **D** requires conformational isomerisation of one of the aryl ligands and subsequent rearrangement of the binding to gold. This rearrangement costs energy, but results in the bullvalene 3-membered ring becoming aligned away from the direction of tension so that

this (rigid) unit does not bare the applied force. The net effect is that **D** becomes destabilised with respect to **A** at larger tip extensions, as is evident from Figure 5b. If other interface structures between the bullvalene and the gold are obtained, then the basic features evident in Supplementary Figure 9 are likely to remain as placing any junction under tension will most likely lead to the shown alignments between the bullvalene atoms and the force direction. Hence the depicted scenario is expected to remain robust, and therefore increasing the **A** to **D** transition-state energy by 0.13 eV presents an unrealistic scenario for interpreting the observed junction properties.

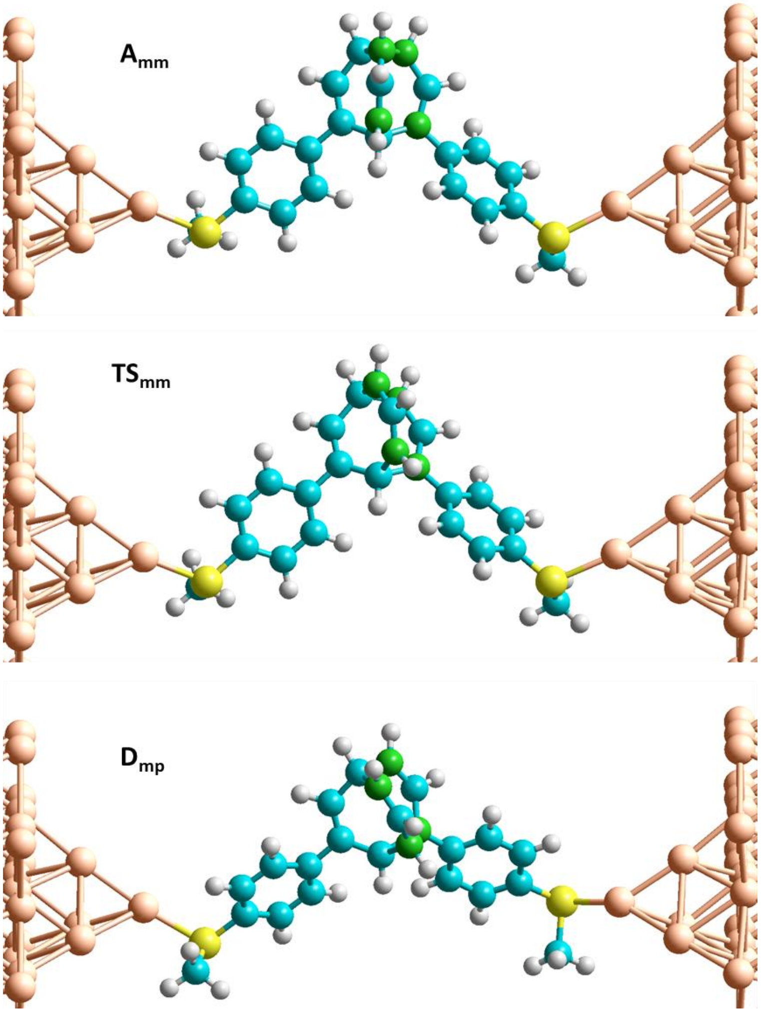

Supplementary Figure 9 | A to D conversion at d = 16.4 Å, showing the reactant, transition state and produce structures with the four carbon atoms involved in the Cope rearrangement highlighted in green (gold- gold, carbon- cyan, sulfur- yellow, hydrogen- white). Source data are provided as a Source Data file.

The alternate empirical scenario considered in which the energy of the **D** conformers is increased by 0.1 eV and the associated transition-state energies increased by 0.05 eV is consistent with the forces apparent in Supplementary Figure 9. This scenario assumes that the calculations underestimate the effect of tension on the three-membered ring in **D**, commensurately underestimating its effect on the **A** to **D** transition-state. It can account for observed conductance properties, except for the observation of most junctions breaking before isomerisation.

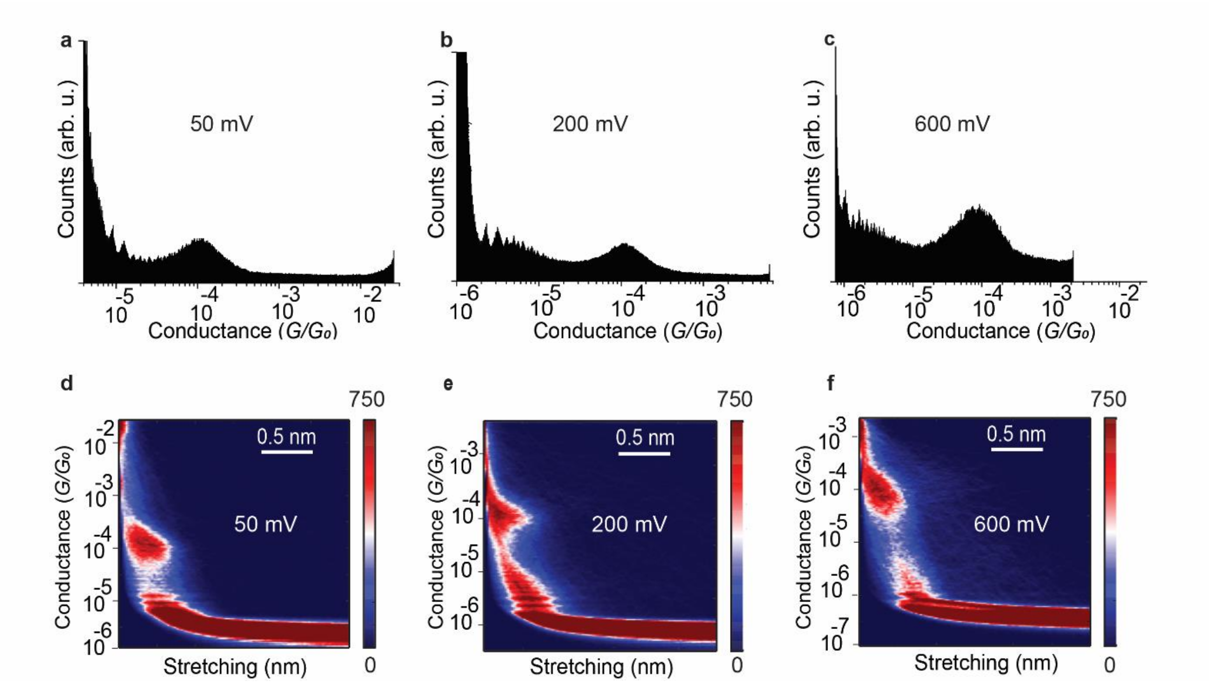

Supplementary Figure 10 | STMBJ conductance histograms at different bias voltages. The signal at ≈ 100 μG_0 remains unchanged with increasing bias, whereas the signals in the region of 1 – 10 μG_0 broaden to extend over a wider conductance range with increasing bias voltage. The histograms in (a and d), (b and e) and (c and f) were all performed without selection. The number of current decays in (a and d) is 5067, in (b and e) 5069 and in (c and f) is 3134. The bias voltage in (a and d) is 50 mV, in (b and e) is 200 mV and in (c and f) is 600 mV. The dark blue color in (d–f) represents the absence of data (0 counts) while the red color represents the maximum counts for accumulated data, as indicated in the color-bar scale.

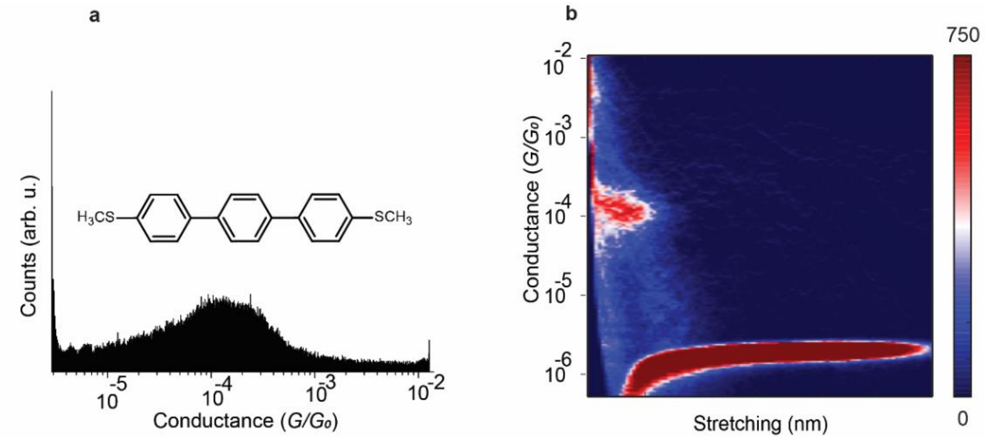

Supplementary Figure 11 | Conductance histogram for a control molecule. The molecule comprises S–CH_3 groups identical to the ones used for the main molecule studied in the manuscript but without the bullvalene center. Hence, the molecule cannot undergo constitutional isomerism or significant internal conformational isomerism, showing only one signal at ca. 150 μG_0. The histograms in (a and b) were performed without any selection procedure. The number of current decays in (a) and (b) is 3200. The dark blue color in (b) represents the absence of data (0 counts) while the red color represents the maximum counts for accumulated data, as indicated in the color-bar scale.

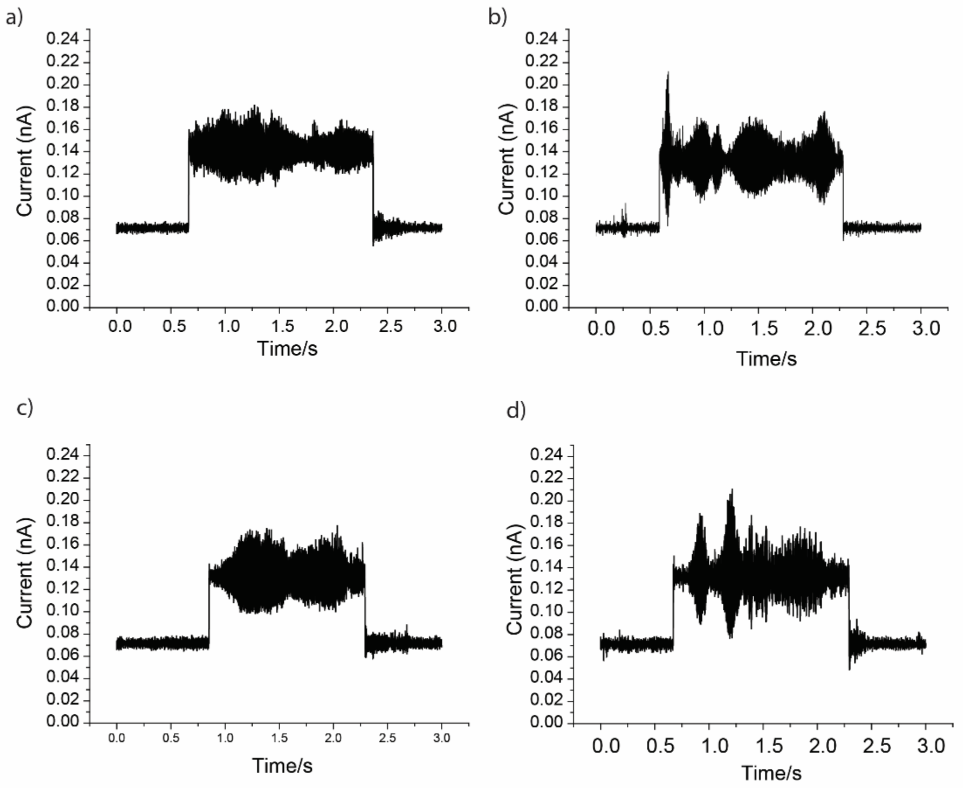

Supplementary Figure 12 | a–d. Represenatative Bullvalene blinks at an electrode–electrode seperation of 16.2 Å. The surface bias was +100 mV. Histograms accumulating the 100 blinks and their FFT analysis are shown in Figure 4 of the main manuscript.

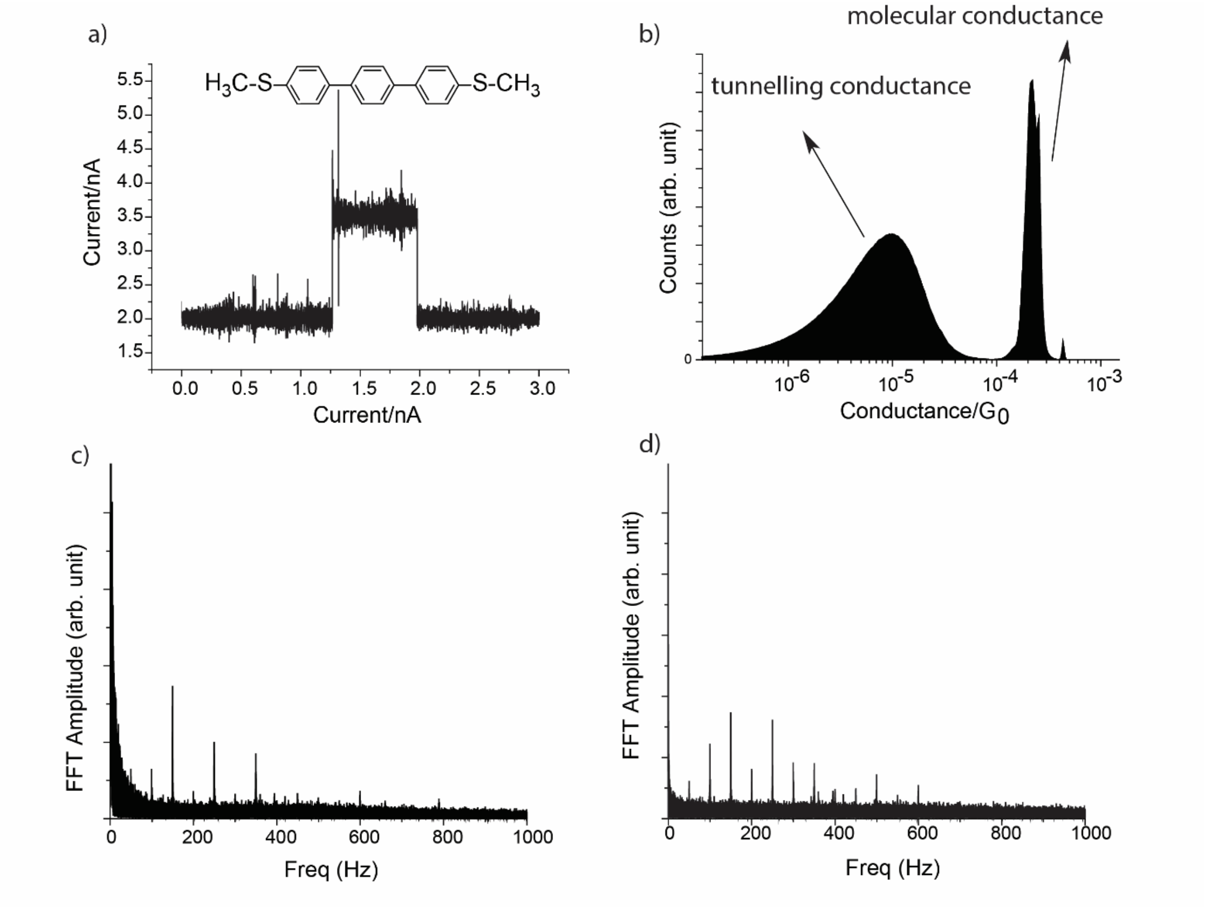

Supplementary Figure 13 | Blinking experiments for the control molecule. (a) A represantative blink obtained at a surface bias of +100 mV. The tunneling distance is 1.25 nm. (b) Conductance histogram accumulating 100 blinks. (c) FFT analysis of the blinks. (d) FFT analysis of the backgoround tunneling current.

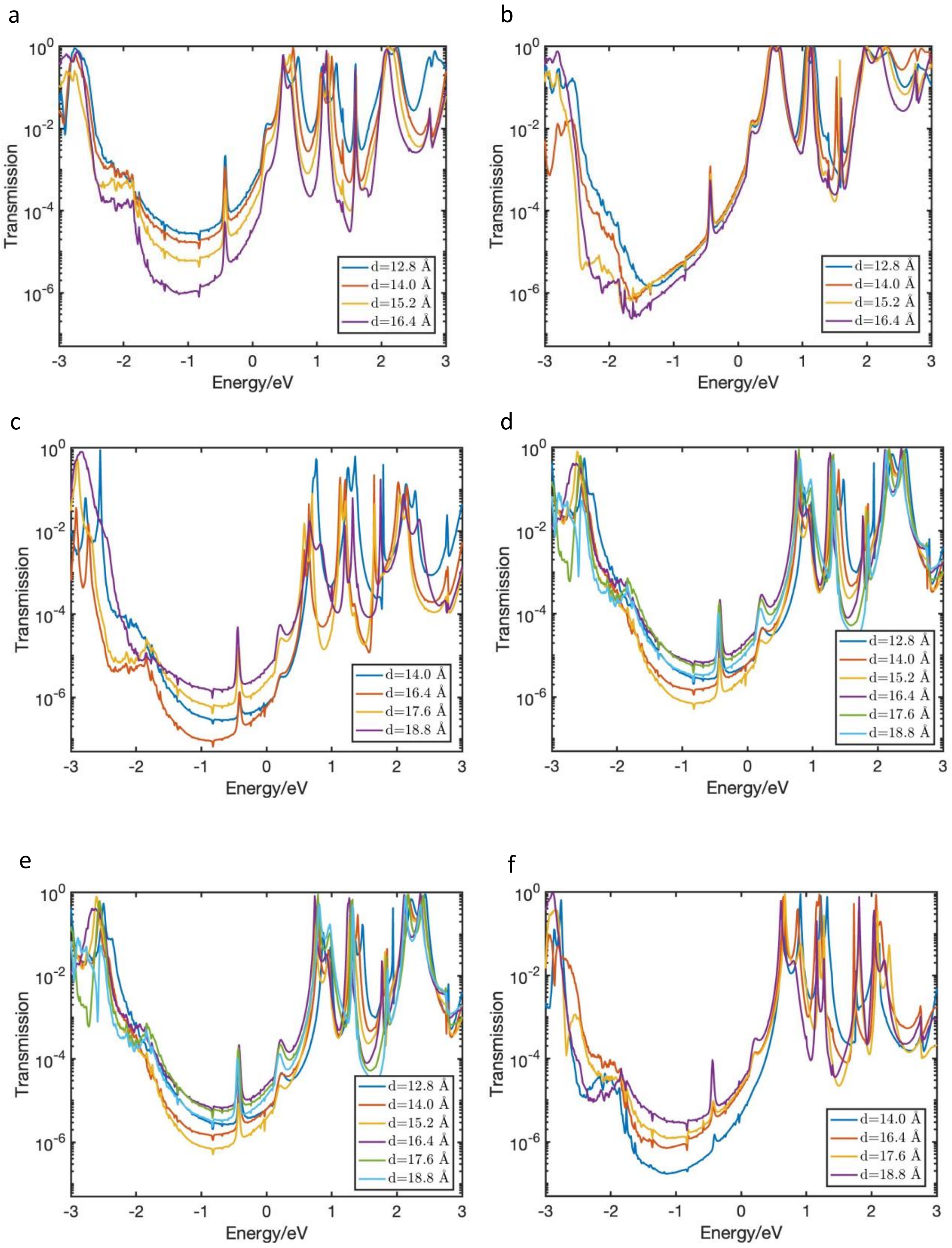

Supplementary Figure 14 | NEGF calculated transmissions through Au-diarylbullvalene-Au linkages. The plot shows transmissions as functions of energy away from the Fermi energy computed for different conformers and stretching distances d: a) $\mathbf{A_{mm}}$, b) $\mathbf{A_{pm}}$, c) $\mathbf{B_{mm}}$, d) $\mathbf{B_{mp}}$, e) $\mathbf{B_{pm}}$, f) $\mathbf{C_{mm}}$.

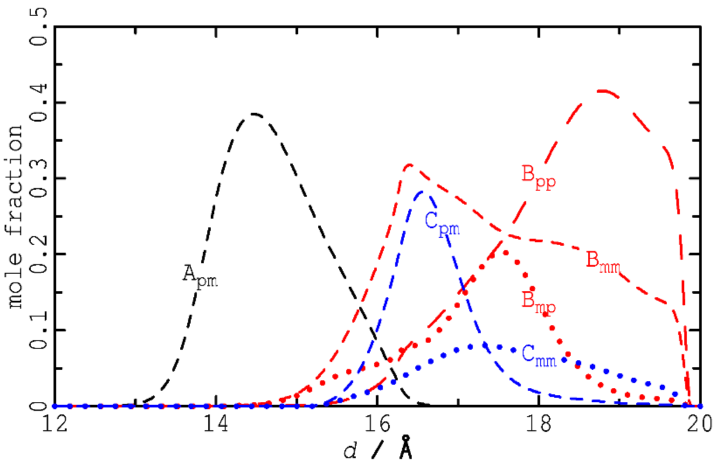

Supplementary Figure 15 | Other mole fractions obtained from the kinetics simulation of retracted 4-atom tips, analogous to the results presented in Figure 6f.

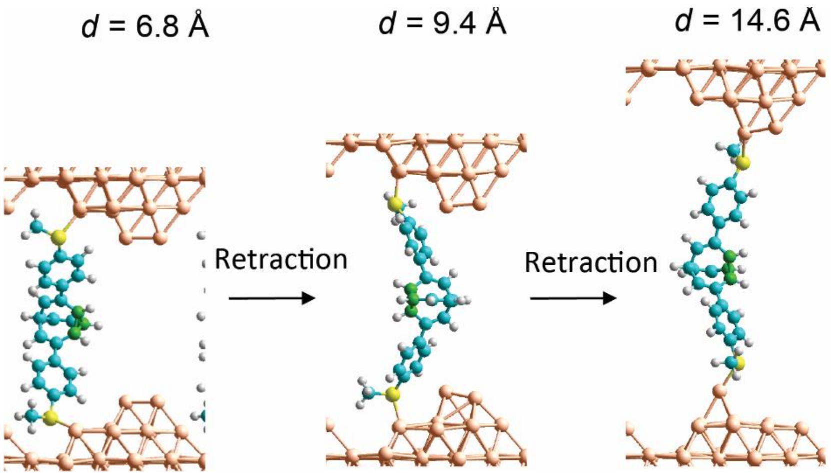

Supplementary Figure 16 | Some configurations passed through during the MD retraction of the restructured tip C_{mp} starting at d = 6.835 Å. Source data are provided as a Source Data file.

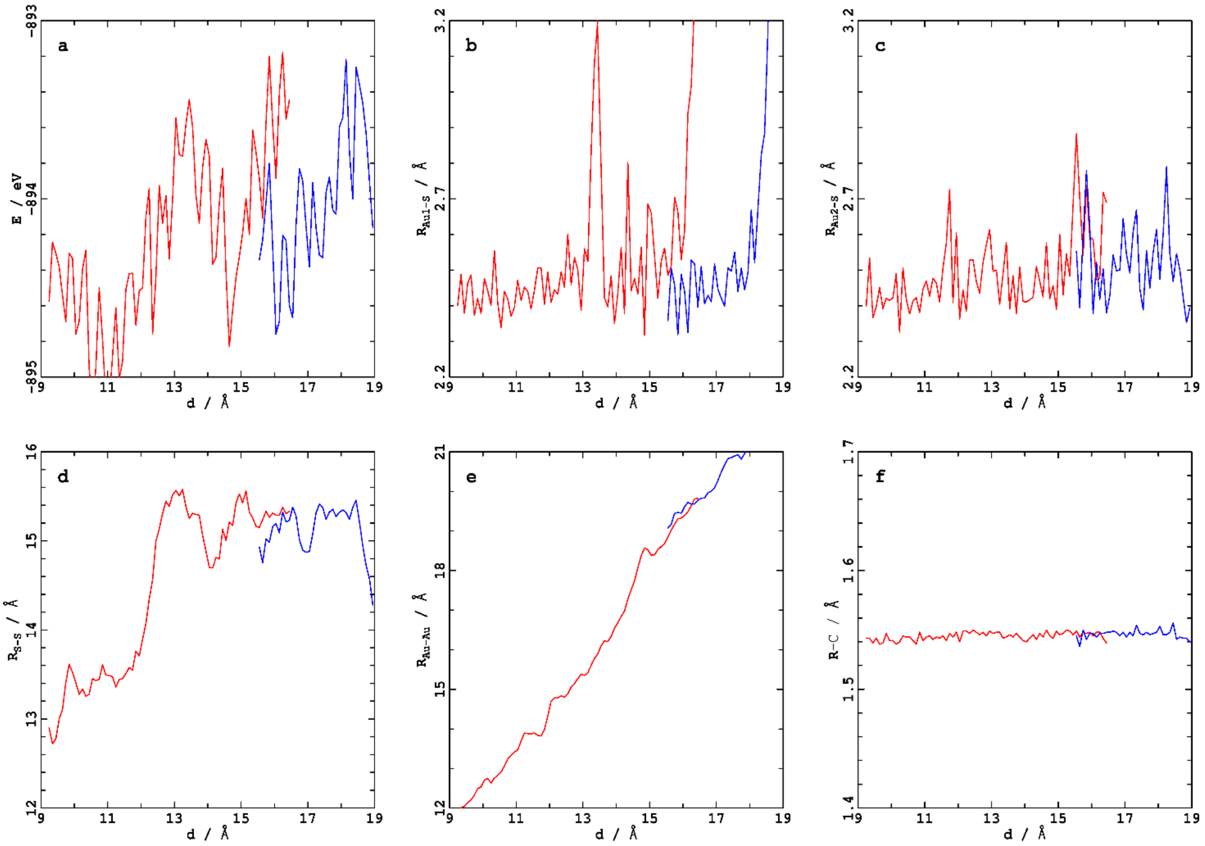

Supplementary Figure 17 | Some properties of the MD simulations of tip retraction for C_{mp} starting at the interface geometry shown in Figure 6e. Each colour represents a continuous MD run, with changes indicating that conformational or interface isomerism was manually enforced. The plots show: (a) the potential energy; (b) and (c) fluctuations in the shortest Au – S bond length; (d) fluctuations in the S – S distance; (e) fluctuations in the shortest inter-tip Au-Au distance; and (f) the average of the three C – C bonds in the bullvalene 3-membered ring. Source data are provided as a Source Data file.

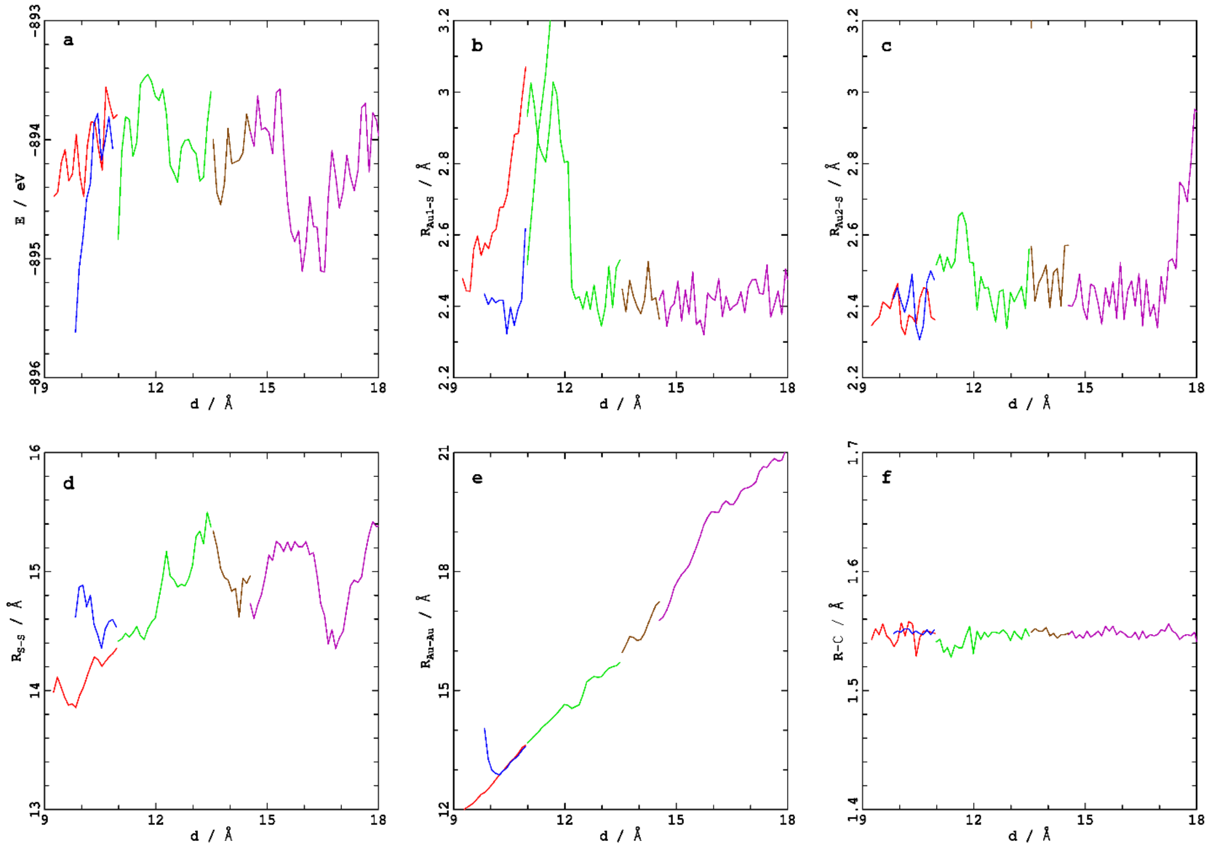

Supplementary Figure 18 | Some properties of the MD simulations of tip retraction for E starting at a structure analogous to the interface geometry for **C_{mp}** shown in Figure 6e. Each colour represents a continuous MD run, with changes indicating that conformational or interface isomerism was manually enforced. The plots show: (a) the potential energy; (b) and (c) fluctuations in the shortest Au – S bond length; (d) fluctuations in the S – S distance; (e) fluctuations in the shortest inter-tip Au-Au distance; and (f) the average of the three C – C bonds in the bullvalene 3-membered ring. Source data are provided as a Source Data file.

Supplementary Table 1 | Calculated properties of diaryl bullvalene constitutional and conformational isomers in the gas phase. Source data are provided as a Source Data file.

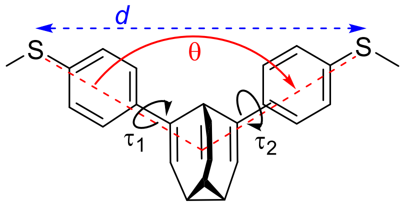

Isomer	θ / °	d / Å	τ_1 / °	τ_2 / °	ΔE / eV
$\mathbf{A_{mm}}$	86.3	10.8	-33.4	-35.4	0.02
$\mathbf{A_{pm}}$	88.6	11.1	34.6	-34.6	0.10
$\mathbf{A_{mp}}$	86.2	10.8	-34.7	34.7	0.03
$\mathbf{B_{mm}}$	106.5	12.7	-40.6	-39.6	0.09
$\mathbf{B_{mp}}$	103.1	12.3	-42.6	42.4	0.00
$\mathbf{B_{pm}}$	102.2	12.3	43.4	-41	0.11
$\mathbf{B_{pp}}$	100.5	12.1	43.8	43.5	0.03
$\mathbf{C_{mm}}$	103.4	12.4	-41.4	-44.7	0.05
$\mathbf{C_{mp}}$	103.4	12.4	-41.1	41.1	0.00
$\mathbf{C_{pm}}$	104.9	12.6	44.9	-44.9	0.12
**D**	90.5	11.1	39.6	73.6	0.22
**E**	139.0	14.7	38.5	83.9	0.20
**F**	54.9	7.0	58.4	-58.8	0.35
**G**	102.9	12.2	41.8	29.7	0.40
**H**	152.4	15.0	-75.4	29.4	0.53
**I**	57.0	7.5	-51.6	13.8	0.49
**J**	55.8	7.1	57.3	69.9	0.31
**K**	103.5	12.0	38.4	84.9	0.20
**L**	60.9	8.0	-50.9	-49.9	0.17

Supplementary Table 2 | Calculated relative energies, in eV, for diaryl bullvalene isomers bound between gold contacts with 4-atom tips, at cluster-model apex separation d.

d / Å	**A_{mm}**	**A_{mp}**	**A_{pm}**	**B_{mm}**	**B_{mp}**	**B_{pm}**	**B_{pp}**	**C_{mm}**	**C_{mp}**	**C_{pm}**	**D**	**E**	**F**	**G**	**H**	**I**	**J**	**K**	**L**
9.2			0.185																
10.4	0.033		0.231	0.297	0.253	0.500	0.466	0.201	0.306	0.285									
11.6	0.011		0.216	0.265	0.264	0.423	0.424	0.216	0.281	0.227									
12.8	[0]	0.169	0.175	0.259	0.273	0.386	0.382	0.240	0.285	0.211	0.505		0.432	0.470		0.358	0.358	0.388	0.356
14.0	0.066	0.073	0.176	0.239	0.251	0.329	0.328	0.255	0.279	0.199	0.505			0.509				0.304	
15.2	0.171	0.178	0.252	0.217	0.224	0.281	0.290	0.245	0.266	0.216	0.389	0.644		0.551	0.770			0.375	
16.4	0.374	0.410	0.395	0.244	0.280	0.285	0.279	0.303	0.283	0.24	0.668	0.507		0.646	0.810			0.397	
17.6	0.795	0.889	0.753	0.282	0.285	0.291	0.282	0.332	0.317	0.33	1.336	0.512		0.825	0.757			0.655	
18.2	1.091	1.214	1.057								1.405								
18.8				0.494	0.545	0.497	0.474	0.548	0.516	0.583		0.558			0.903				
20.0				0.990	1.064	1.003	0.968	1.059	1.014	1.110		1.004							

Supplementary Table 3 | Calculated transition-state energies, in eV, relative to the isomer energies listed in Supplementary Table 2, for diaryl bullvalene isomers bound between gold contacts with 4-atom tips, at cluster-model apex separation d.

d / Å	$\mathbf{A_{mm} – D_{mm}}$	$\mathbf{B_{mm} – C_{mm}}$	$\mathbf{B_{mm} – D_{mm}}$	$\mathbf{B_{mm} – E_{mm}}$	**D – G**	**G – K**
14.0	0.512		0.632			
15.2	0.578	0.712	0.659		0.910	0.837
16.4	0.801	0.716	0.781	0.749	0.969	0.901
17.6	1.190	0.764	1.029	0.736	1.215	1.173
18.2	1.405		1.405			
18.8		0.936		0.883		
20.0		1.461		1.330		

Supplementary Table 4 | Calculated conductance, in μG_0, for diaryl bullvalene isomers bound between gold contacts with 4-atom tips, at cluster-model apex separation d.

d / Å	$\mathbf{A_{mm}}$	$\mathbf{A_{mp}}$	$\mathbf{A_{pm}}$	$\mathbf{B_{mm}}$	$\mathbf{B_{mp}}$	$\mathbf{B_{pm}}$	$\mathbf{B_{pp}}$	$\mathbf{C_{mm}}$	$\mathbf{C_{mp}}$	$\mathbf{C_{pm}}$	**D**	**E**
9.2		2100										
10.4	171	199										
11.6	354	496										
12.8	513.5	429.2	3.3	0.8	6.1	29	2	13	3.5	4.5	139	
14.0	351.6	524.3	0.9	0.6	5.8	44.1	2.8	4.5	6.3	3.9	189	
15.2	148.4	444.7	0.6	0.6	3.3	64.8	2.9	3.3	16	2.6	3.1	2.6
16.4	39.6	280.2	1	0.5	24.7	45.8	3	24.1	195.6	1.3	1.5	124
17.6	10	121	1	2.5	18.7	53.5	1.1	26.6	523.7	0.5	7	111
18.2	4.8	65.8	2								5	
18.8				5.2	12.9	90	1.4	31.2	629.6	0.5		140
20.0				1.5	123	129	2.5	26.2	342	18.2		8.9

Supplementary Table 5 | Relative energies calculated for different diaryl bullvalene isomers bound to gold contacts in the configuration shown in Figure 7e shortened by one Au-layer spacing to d = 6.835 Å, along with their conductance calculated using NEGF theory. Source data are provided as a Source Data file.

Isomer	ΔE / eV	G / μG_0
$\mathbf{A_{mm}}$	0.25	2.2
$\mathbf{A_{mp}}$	0.29	1.5
$\mathbf{A_{pm}}$	0.26	1.6
$\mathbf{A_{pp}}$	0.28	44.8
$\mathbf{B_{mm}}$	0.38	3.9
$\mathbf{B_{mp}}$	0.34	18.4
$\mathbf{B_{pm}}$	0.29	18.3
$\mathbf{B_{pp}}$	0.05	1.9
$\mathbf{C_{mm}}$	0.15	50.2
$\mathbf{C_{mp}}$	0.13	63.6
$\mathbf{C_{pm}}$	0.00	10.2
$\mathbf{C_{pp}}$	0.07	28.6
**D**	0.36	1.3
**E**	0.00	73.3
transition-state energies		
$\mathbf{A_{mm}}$ – $\mathbf{D_{mm}}$	0.81	
$\mathbf{B_{mm}}$ – $\mathbf{C_{mm}}$	0.85	
$\mathbf{B_{mm}}$ – $\mathbf{D_{mm}}$	0.84	
$\mathbf{B_{mm}}$ – **E**	0.62	

Supplementary References

1 Aragonès, A. C. *et al.* Control over Near-Ballistic Electron Transport through Formation of Parallel Pathways in a Single-Molecule Wire. *Journal of the American Chemical Society* **141**, 240-250, doi:10.1021/jacs.8b09086 (2019).
