# Supplementary material for: Controlling piezoresistance in single molecules through the isomerisation of bullvalenes
Source: Nat Commun. 2023 Oct 3;14:6089. doi: 10.1038/s41467-023-41674-z (PMC10547723; doi:10.1038/s41467-023-41674-z)
Supplement: Supplementary file 3 — Reporting Summary [file 41467_2023_41674_MOESM3_ESM.pdf]

nature portfolio

Corresponding author(s): Nadim Darwish

Last updated by author(s): 17-08-2023

Reporting Summary

Nature Portfolio wishes to improve the reproducibility of the work that we publish. This form provides structure for consistency and transparency in reporting. For further information on Nature Portfolio policies, see our Editorial Policies and the Editorial Policy Checklist.

Statistics

For all statistical analyses, confirm that the following items are present in the figure legend, table legend, main text, or Methods section.

n/a	Confirmed	
☐	☒	The exact sample size (*n*) for each experimental group/condition, given as a discrete number and unit of measurement
☒	☐	A statement on whether measurements were taken from distinct samples or whether the same sample was measured repeatedly
☒	☐	The statistical test(s) used AND whether they are one- or two-sided<br>*Only common tests should be described solely by name; describe more complex techniques in the Methods section.*
☒	☐	A description of all covariates tested
☒	☐	A description of any assumptions or corrections, such as tests of normality and adjustment for multiple comparisons
☒	☐	A full description of the statistical parameters including central tendency (e.g. means) or other basic estimates (e.g. regression coefficient) AND variation (e.g. standard deviation) or associated estimates of uncertainty (e.g. confidence intervals)
☒	☐	For null hypothesis testing, the test statistic (e.g. *F*, *t*, *r*) with confidence intervals, effect sizes, degrees of freedom and *P* value noted<br>*Give P values as exact values whenever suitable.*
☒	☐	For Bayesian analysis, information on the choice of priors and Markov chain Monte Carlo settings
☒	☐	For hierarchical and complex designs, identification of the appropriate level for tests and full reporting of outcomes
☒	☐	Estimates of effect sizes (e.g. Cohen's *d*, Pearson's *r*), indicating how they were calculated

Our web collection on statistics for biologists contains articles on many of the points above.

Software and code

Policy information about availability of computer code

Data collection	STM break junction data was collected using a custom made algorithm written in Labview from National Instruments. The program requires specific training before it can be used. We are happy to provide the code and explanations on how to use it upon reasonable request.
Data analysis	STM break junction data were analysed using a custom made algorithm written in Labview from National Instruments. The program requires specific training before it can be used. We are happy to provide the code and explanations on how to use it upon reasonable request. The kinetics Monte Carlo simulation code and data are available on Zenodo.

For manuscripts utilizing custom algorithms or software that are central to the research but not yet described in published literature, software must be made available to editors and reviewers. We strongly encourage code deposition in a community repository (e.g. GitHub). See the Nature Portfolio guidelines for submitting code & software for further information.

Data

Policy information about availability of data

All manuscripts must include a data availability statement. This statement should provide the following information, where applicable:

- Accession codes, unique identifiers, or web links for publicly available datasets
- A description of any restrictions on data availability
- For clinical datasets or third party data, please ensure that the statement adheres to our policy

The data that support the findings of this study are available from the corresponding authors upon request. STM experimental data is available from N.D. upon request. To ensure proper accessibility, the data will be converted from a binary format to a text format. We will be pleased to perform this conversion along with

providing the necessary calibration specifications upon request. Synthetic methods and NMR spectra are available from T.F. NEGF calculation results are available from D.K. Source data are provided with this paper.

Research involving human participants, their data, or biological material

Policy information about studies with human participants or human data. See also policy information about sex, gender (identity/presentation), and sexual orientation and race, ethnicity and racism.

Reporting on sex and gender	NA
Reporting on race, ethnicity, or other socially relevant groupings	NA
Population characteristics	NA
Recruitment	NA
Ethics oversight	NA

Note that full information on the approval of the study protocol must also be provided in the manuscript.

Field-specific reporting

Please select the one below that is the best fit for your research. If you are not sure, read the appropriate sections before making your selection.

☒ Life sciences ☐ Behavioural & social sciences ☐ Ecological, evolutionary & environmental sciences

For a reference copy of the document with all sections, see nature.com/documents/nr-reporting-summary-flat.pdf

Life sciences study design

All studies must disclose on these points even when the disclosure is negative.

Sample size	For STMBJ data, the sample size was specified in the captions under each figure. Typically the sample size is above 2500 current-distance curves. This procedure leads to clear conductance peaks in the 1D and 2D histograms.
Data exclusions	for STMBJ data, all data were included in performing the histograms otherwise specified if some curves were rejected. In that case, the complete set is shown in the Supplementary Information with out any data rejected.
Replication	The STMBJ data is a repetitive type of experiments in which the STM tip is driven in and out from a surface in the presence of a dilute solution of the molecules of interest. The number of single molecule junctions performed to produce each histogram is specified in the figure captions.
Randomization	Not applicable as there were no participants or subjects being assigned to different groups or conditions.
Blinding	Not applicable in our study since there were no participant subjects (human or animal) involved. The possibility of bias in participant behaviour is therefore not relevant.

Reporting for specific materials, systems and methods

We require information from authors about some types of materials, experimental systems and methods used in many studies. Here, indicate whether each material, system or method listed is relevant to your study. If you are not sure if a list item applies to your research, read the appropriate section before selecting a response.

Materials & experimental systems

n/a	Involved in the study	
☒	☐	Antibodies
☒	☐	Eukaryotic cell lines
☒	☐	Palaeontology and archaeology
☒	☐	Animals and other organisms
☒	☐	Clinical data
☒	☐	Dual use research of concern
☒	☐	Plants

Methods

n/a	Involved in the study	
☒	☐	ChIP-seq
☒	☐	Flow cytometry
☒	☐	MRI-based neuroimaging
